# A Randomized, Controlled Clinical Trial of Combining Therapy with Traditional Chinese Medicine-Based Psychotherapy and Chinese Herbal Medicine for Menopausal Women with Moderate to Serious Mood Disorder

**DOI:** 10.1155/2019/9581087

**Published:** 2019-01-06

**Authors:** Yuyan Zeng, Xuchun Huang, Changqian Chen, Guangning Nie, Xiaojing Cao, Jiju Wang, Xiaoyun Wang

**Affiliations:** ^1^Department of Gynecology, The Second Affiliated Hospital of Guangzhou University of Traditional Chinese Medicine, Guangzhou 510120, China; ^2^Guangzhou University of Traditional Chinese Medicine of Postdoctoral Scientific Research, Guangzhou 510120, China; ^3^China Integrated Chinese and Western Medicine Hospital of Zhuhai, Zhuhai 510120, China; ^4^The Second Clinical Medicine School of Guangzhou University of Chinese, Guangzhou 510120, China

## Abstract

**Objective:**

To comparatively examine the effectiveness and safety of the combination therapy of traditional Chinese medicine formula Bushen-Shugan granule and psychotherapy (BSSG-P) and Chinese herbal medicine Bushen-Shugan granule (BSSG) alone in the treatment of moderate to serious mood disorder in menopausal women.

**Methods:**

In our previous clinical studies, BSSG-P had been proved to be superior to BSSG, psychological treatment, and placebo in improving mild mood disorder in menopausal women. In this study, we analyzed the efficacy of BSSG-P and BSSG in the treatment of moderate to serious mood disorder. Eighty-five eligible participants, who were diagnosed as menopausal women with moderate to serious mood disorder and categorized as kidney deficiency and liver-qi stagnation pattern, were randomly assigned into two groups and treated with BSSG-P or BSSG. They were subjected to an 8-week treatment period and a 4-week follow-up study. The primary outcome instrument was the Greene Climacteric Scale, Self-Rating Depression Scale (SDS), and Self-Rating Anxiety Scale (SAS), respectively.

**Results:**

When comparing all time points with baseline, both BSSG-P and BSSG markedly decreased the total score of Greene, SDS, and SAS and the score of each dimension, in which BSSG-P exerted superior effect after 8-week treatment and 4-week follow-up (*P*<0.05). Furthermore, BSSG-P also showed great advantage in reducing the score of Greene, SDS, and SAS for menopausal women with moderate mood disorder at the end of the 8th and 12th week when compared with BSSG (*P *< 0.05), whereas there was no significant difference between groups at any time point for patients with serious mood disorder (*P*>0.05). No serious event occurred in both groups, and no significant difference was found between groups in adverse event proportion.

**Conclusions:**

BSSG-P was superior to BSSG in improving the physical and psychological symptoms of menopausal women with mood disorder. For patients with moderate mood disorder, BSSG-P showed obvious advantages; however, no superiority was observed for serious mood disorder.

## 1. Introduction

Perimenopausal syndromes (PMS) are a group of symptoms that refer to autonomic nervous dysfunction in women before and after menopause caused by ovarian function decline, pituitary hyperfunction, fluctuation, or decline of estrogen level, accompanied by neuropsychological symptoms, also known as climacteric syndrome. In this phase, many women suffer from physical and psychological symptoms such as hot flashes, night sweats, menstrual irregularities, depression, anxiety, nervous tension, and others [1]. Although the menopausal transition is a natural part of women's reproductive life course, it usually affects quality of life and mood. Mood disorder in menopausal women is a nonspecific clinical syndrome characterized by anxiety and depression before and after natural menopause, and the prevalence of depressive symptoms in menopausal women is 5.9%-23.8% [2–5]. Women in the early menopause transition were 2-3 times more likely to suffer from depression than those in premenopause, and the risk increased to 14 times in the late menopause transition [6–8]. Recent longitudinal epidemiological studies [4, 9] indicate that women's experience of depressive and anxiety symptoms was higher in the menopausal transition than other periods. It is known that the most widely used treatment is hormone therapy (HT). Early intervention with a moderate dose of HT could alleviate some perimenopausal syndromes. However, its side effects compromise its clinical application. HT is not applied to those with unexplained vaginal bleeding, active liver disease, coronary heart disease, personal history of thromboembolic disease, etc. It is also associated with risk of stroke, myocardial infarction, and breast cancer [10–12]. Menopause hormone therapy (MHT) may be beneficial for perimenopausal women depression but antidepressant therapy is still the first-line treatment [13]. Disappointingly, the efficacy of treatment with antidepressant drugs is not obvious and requires long-term use to form dependence. Thus, both the classic HT and antidepressant therapy cannot relieve all the symptoms that may present in patients. Furthermore, a Menopause Matters survey of 1,633 women showed that more than half of women do not like to use HT [14]. On the contrary, 22.1% of women favor the use of Chinese herbal medicine (CHM) to manage their menopausal symptoms in the United States [15]. Therefore, a safe and effective alternative therapy for the treatment of menopausal symptoms is urgently needed.

Our research group has previously conducted a lot of clinical studies. The results showed that TCM-based psychotherapy combined with CHM could significantly improve the menopausal syndrome and was superior to CHM and the placebo group, with a stable, lasting, and safe curative effect [16, 17].

Bushen-Shugan (BSSG) granule is proposed by Professor Xiao-yun Wang, who is an academic leader of the second Affiliated Hospital of Guangzhou University of Chinese Medicine and has rich experience in treating menopausal symptom with CHM and TCM-based psychotherapy. BSSG has been applied in the clinic for years. Combination therapy with Bushen-Shugan granules-based psychotherapy (BSSG-P) is a method for treating interaction between traditional Chinese medicine and traditional psychotherapy. It is based on TCM theory and experiences and has been widely used for the treatment of many diseases, especially for those associated with psychological symptoms, such as menopausal syndrome [18]. Our preliminary study has showed that BSSG-P was effective in the treatment of mild mood disorder in menopausal women. As a follow-up study, in the present work, we optimized and developed the treatment methods of BSSG-P on the basis of previous researches and endeavored to evaluate its potential effectiveness and safety for menopausal women with moderate to serious mood disorder. Then we sought to investigate whether different degrees of mood disorder had the same sensitivity to the same therapeutic approach or which would be better for a certain degree of mood disorder. Finally, we hoped to set up supporting evidence for TCM-based psychotherapy combined with CHM in the treatment of menopausal mood disorder.

## 2. Methods

### 2.1. Study Design

A randomized, controlled trial was designed and conducted by the researchers from DME center of Guangzhou University of Traditional Chinese Medicine. Subjects were assigned to BSSG-P and BSSG group at 1:1 ratio according to random numbers produced by statistical software. Our preliminary study showed that BSSG-P group could significantly improve the menopausal syndrome when compared with BSSG, psychological group, and placebo group [19]; therefore, we just compared the curative effect of BSSG-P and BSSG group in this study. The results of random distribution shall be stored in duplicate in the form of a file, and the method, process, group setting, and grouping results of the recorded random numbers shall be explained and recorded for inspection when necessary. A blinded design could not be conducted because the trial included BSSG-P group. To try to make the results more equitable, the efficacies were assessed by a third party who was unaware of the interventions.

### 2.2. Participants and Setting

85 cases of women aged 41-60 years with moderate to serious mood disorder in menopausal women were selected in the gynecological outpatient department of the second Affiliated Hospital of Guangzhou University of Chinese Medicine from February 2016 to January 2017. The clinical trial followed the principles of the Declaration of Helsinki and the guidelines of Good Clinical Practice and had obtained the approval from the Medical Ethics Review Committee of the second Affiliated Hospital of Guangzhou University of Chinese Medicine. The clinical trial registration code is ChiCTR-IOR-16007639. All the participants fully understood the procedure and signed informed consent.

#### 2.2.1. Diagnostic Criteria

According to the guidelines for clinical diagnosis and treatment-obstetrics and gynecology booklet prepared by Chinese Medical Association (published by People's Medical Publishing House of China in 2011), all patients suffered from menstrual disorders or amenorrhea, simultaneously with vasomotor instability symptoms, psychiatric symptoms and symptoms of urogenital atrophy were diagnosed menopausal syndrome. Organic lesions should be eliminated by gynecological examination or pelvic ultrasound examination.

The TCM Syndrome Diagnostic Criteria light the guidelines for the diagnosis and treatment of gynecological diseases issued by the China Association for Traditional Chinese Medicine in TCM Symptoms of Menopausal syndrome [20]. The main symptoms include hot sweat out, abnormal moods (including irritability/testiness, depression, joys, and sorrows). The secondary symptoms contain soreness of waist, breast swelling, abdominal distension after eating, insomnia and wakefulness, light red tongue, thin white tongue coating, thinness and string pulse or thinness, and sunken pulse. Patients with all of the main symptoms and at least 2 of the secondary symptoms could be identified to be kidney deficiency and liver-qi stagnation pattern. The diagnosis should be completed by 2 separate gynecological physicians.

#### 2.2.2. Inclusion Criteria

1. Aged 41 to 60 years.
2. Accord with diagnostic criteria for menopausal symptom and kidney deficiency and liver-qi stagnation of TCM syndrome.
3. FSH greater than 10 IU/L.
4. According to the mental illness diagnosis and statistics manual DSM-iv, patients whose depression/anxiety symptoms occur within 3 years of menopause or perimenopause symptoms, or within 5 years of menopause, and these symptoms cannot be explained by other physical and mental disorders are diagnosed with depression or anxiety disorders. Moreover, these symptoms persist for at least 30 days prior to screening.
5. Moderate to serious mood disorder: Self-Rating Depression Scale (SDS) ≥ 62 and/or Self-Rating Anxiety Scale (SAS) ≥ 62 (standard score) (total score of the scale × 1.25 = standard score). Moderate mood disorder: 62 ≤ SDS ≤ 72 or 62 ≤ SAS ≤ 72, serious mood disorder: 73 < SDS < 85 or 73 < SAS < 85.
6. Volunteered to participate in this study and signed informed consent.

#### 2.2.3. Exclusion Criteria

All patients with the other mood disorders including mental illnesses, schizophrenia, obsessive compulsive disorder, etc., agnogenic vaginal anomalous bleeding, ovarian neoplasms, bilateral oophorectomies, participation of phase I and II clinical trials in the past 6 months or phase III and IV clinical trials in 3 months, hypersensitive constitution or allergies to the medicines, suspicious or definite precancerous lesions of the cervix, breast cancer, severe primary diseases of the cardiovascular system, cerebral vessels, liver, kidney, or hematopoietic system, uncontrolled or untreated hypertension, uncontrolled or untreated diabetes; or abnormal thyrotropin which may cause symptoms similar to menopause were excluded from this trial.

### 2.3. Interventions

*(1) Chinese Herbal Medicine. *BSSG, composed of prepared rehmannia root (Shudi-Huang in Chinese), radix curcumae (Yu-Jin), and peucedanum praeruptorum (Qian-Hu), etc. (this prescription dose of Chinese traditional medicine boiling-free granules was converted from the dosage of Chinese medicine herbal, and the effectiveness of granules with separate wrapping, controlled quality, and optimum dosage was certified as being equal to or better than the herbal [21, 22]), was provided by Jiangyin Jiangtian (Jiangsu province of China) Pharmaceutical Co. Ltd. The company is a traditional Chinese medicine formula particle pilot production enterprises which has passed the national GMP certification and has been approved by the State Food and Drug Administration. BSSG was orally taken in packs twice a day. The treatment duration was 8 weeks and followed up for 4 weeks.

*(2) Intervention and Optimization of TCM-Based Psychotherapy Combined with BSSG. *A bright, warm, quiet room with 6 to 10 soft and comfortable seats must be provided for psychotherapy, in which there was equipped with soft light, DVD device capable of watching comedy, and equipment to respond to emergencies during psychotherapy. In addition, a physician and a nurse with psychological expertise and professional skills as well as knowledge of emergency procedures were involved in this psychotherapy.

In this study, the emotional treatment program was adjusted and improved according to the results of our previous clinical research. The optimization plan was as follows:* the first step* is psychocommunication. Physician induced patients to pour out their feelings and understood the mental cause of diseases for 30 minutes in the first week.* The second step *is induction of the catharsis. Right after the first step, the patients should be guided to vent a strong sad mood and be aroused to cry by displaying a tragedy for once a week in the second and third weeks. TCM theory holds that sorrow could overcome anger and this step can be conducive to get rid of unhealthy feelings.* The third step *is induction of the positive effect of emotion: the patients were exposed to comedy films at the fifth and sixth weeks and induced to laugh and balance their bad emotions and thus was the therapeutic effect of “happiness defeated sorrow” from the perspective of TCM emotional treatment according to TCM theory.

### 2.4. Quality Control of the Study

For assurance of quality control of the study, all researchers involved in this trial accomplished requirements that were trained in the standard operating procedures (SOPs) for TCM-based psychotherapy and were well familiar with efficacy and safety of medicine used in this trial prior to the start. Verifying the test value of significant abnormalities, if the results of the review were still abnormal, the seasons must be traced by the doctor to participate in the clinical trial to do the necessary instructions. The quality controller carried out regular quality control on the subject. The test records and reports were accurate and complete and ensured that the rights and interests of the subjects were protected. The medicine was kept by designated persons and counters and registered every time they were taken. Compliance of patients was investigated by counting the number of remaining drugs.

### 2.5. Sample Size Calculation

On the basis of our previous treatment effect of patients with mood disorder in menopausal women, we expected that the optimized BSSG-P group would reduce the Greene scale by 20 ± 7.52 points and the BSSG group could reduce 14.87 ± 7.90 points. Alpha was 0.05 and the test efficiency was 0.80; we used PEMS 3.1 for windows (Chinese medical encyclopedia/medical statistics) statistical software package, 36 samples were obtained in each group, and 72 samples were needed in both groups by calculation. Allowing for a dropout rate of 15% and taking into account the randomized block design, the required sample size was 71/(1 - 0.15) = 85 cases.

### 2.6. Statistical Analysis

If the measurement data followed the requirements of normality and variance uniformity, the mean of the two samples shall be compared by t test and the comparison before and after treatment used paired t test. When the requirements could not be satisfied, the comparison of the average of the two samples was checked by and the comparison before and after treatment used matched-pairs rank-sum test. The measurement data was checked by normal test when analyzing the clinical characteristics of the two groups before and after treatment. All results with* P*< 0.05 were defined as statistically.

### 2.7. Outcome and Measurements

The Greene Climacteric Scale, composed of 21 items and 5 dimensions, is widely used as a climacteric index or as a quality of life measure for early assessment of menopause syndrome [23–25]. It has rich content and can quickly and intuitively understand the psychological and physical symptoms of menopausal women. Self-Rating Depression Scale (SDS) has 20 projects, each of which is divided into 4 grades. It can directly reflect subjects' subjective feelings, with high reliability and repeatability. Self-Rating Anxiety Scale (SAS) can accurately reflect and differentiate the subjective feelings of anxious psychopaths and healthy people.

The secondary measurement of efficacy included serum levels of dopamine (DA) and norepinephrine (NA).

### 2.8. Safety Assessment

Safety evaluations included thyroid stimulating hormone (TSH), follicle-stimulating hormone (FSH), fasting blood glucose, and cervical pap smear before treatment. And the heart rate, blood pressure, blood and urine routine, hepatic and renal function examination, blood lipid, electrocardiogram, and gynecological ultrasound were detected before and after treatment. Additionally, any adverse events were reported at each clinic visit and recorded on a detailed form.

## 3. Results

### 3.1. Participants and Follow-Up

**Figure 1:**
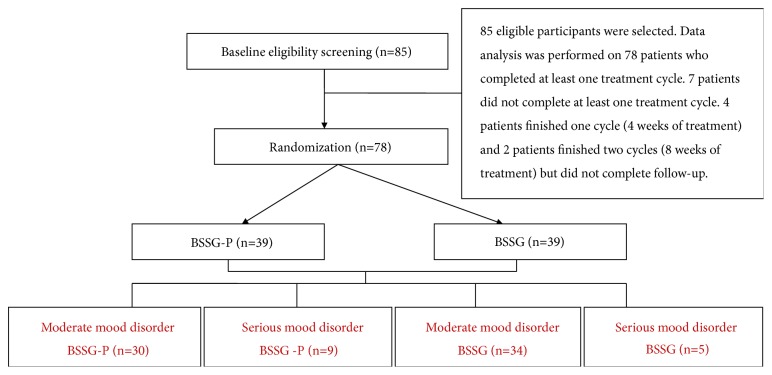
Flow of participants. BSSG-P; BSSG.

In this study, 85 eligible participants were selected and 13 patients shed off, of which 7 cases completed the observation before treatment, 4 patients finished 1 cycle (4 weeks of treatment), and 2 patients finished 2 cycles (8 weeks of treatment). Data analysis was performed on 78 patients who completed at least one treatment cycle (Figure 1).

Baseline demographic and clinical characteristics of participants were comparable between groups. There was no significant difference in terms of age, weight, career, marriage, job stress, smoking, drinking, coffee, menstruation, course of illness, and previous treatment between BSSG-P and BSSG group (*P* > 0.05) (Table 1). No significant difference was found in the baseline data between the two groups of Greene, SDS, SAS, and all the dimensions (*P* > 0.05) (Table 2).

BSSG-P and BSSG for moderate to serious mood disorder in menopausal women.

### 3.2. Primary Outcome

In this study, data statistics followed the principle of Intention-to-Treat (ITT) that was the planned treatment process, rather than based on the actual treatment measures, so that we could better evaluate the treatment effect. Therefore, the ITT of the complete analysis set was used to remove subjects from all randomized subjects in a minimum and reasonable way, and the observed values of the unfinished data were transferred to the last observed value with the last observation.

#### 3.2.1. Comparison of the Total Score of Greene and the Scores of Each Dimension

Through a comprehensive analysis of Greene in menopausal participants, statistical significance could be seen in the Greene total score, anxiety dimension, depression dimension, somatic dimension, and vascular systolic dimension at the end of the 4th, 8th, and 12th week compared with baseline in both BSSG-P and BSSG groups (*P* < 0.05), indicating that each group can improve the quality of life. Impressively, BSSG-P appeared to be more effective treatment for Greene total score, anxiety dimension, depression dimension, and somatic dimension at the end of the 8th week and had better effect on improving Greene total score, anxiety dimension, depression dimension, somatic dimension, and vasoconstriction dimension at the end of the 12th week of follow-up than that of BSSG (*P* < 0.05). However, no statistically significant difference was observed in sexual dimension both in BSSG-P and BSSG group when compared each time point to baseline (*P* > = 0.05), although the BSSG-P group showed an obvious superiority after 4-week treatment (*P* = 0.05). Similarly, there was no significant difference in the total score of Greene and the scores of all dimensions between groups at the end of the 4th week (*P* > 0.05), indicating that the curative effect of these two treatments was equivalent (Table 3).

#### 3.2.2. Comparison of the Total Score of SDS and the Scores of Each Dimension

After the 4th and 8th weeks of treatment and the 12th week of follow-up, both BSSG-P and BSSG could significantly reduce the total score of SDS as well as the scores of anxiety dimension, self-loss dimension, motivation lack dimension, and physiological symptom dimension when compared with those in baseline (*P* < 0.05). Surprisingly, we found that BSSG-P had a better effect on improving the total score of SDS, motivation lack dimension, and anxiety dimension at the end of the 8th and 12th weeks (*P* < 0.05). In addition, effect of ameliorating self-loss dimension was more remarkable in the BSSG-P group than in the BSSG group at the end of the 12th week (*P* < 0.05). But no significant difference was observed between groups in the physiological symptom dimension at any time points (*P*>0.05) (Table 4).

#### 3.2.3. Comparison of the Total Score of SAS and the Scores of Each Dimension

At the end of the 4th, 8th, and 12th week, both BSSG-P and BSSG were effective on dropping decreasing gradients of scores in anxiety dimensions, the dimension of the autonomic nervous disorder, somatic dimension, and exercise tension dimension as well as the total score of SAS when compared with those in baseline (*P*<0.05), in which the effects of total score, anxiety dimension, the dimension of the autonomic nervous disorder, and somatic dimension for BSSG-P treatment were higher than that of BSSG at the end of the 8th and 12th week (*P* < 0.05); however, no significant difference was found between this two groups in exercise tension dimension (*P* > 0.05). After 4 weeks of treatment, the effects of BSSG-P and BSSG were similar in improving SAS score and scores of each dimension, and there was no significant difference between groups in improving exercise tension dimension at any time points (*P* > 0.05) (Table 5).

#### 3.2.4. Comparison of Mean Difference of Greene, SDS, and SAS between Groups

In the foregoing results in this paper we confirmed that treatment of BSSG-P for 4 weeks had no significant advantage when compared to BSSG, so we just analyzed the therapeutic effects for the 8th and 12th week. Results showed that the mean difference (pretreatment minus posttreatment) of Greene, SDS, and SAS in BSSG-P group before and after intervention were more obvious than that of BSSG group after 8 weeks of treatment and at the 12th week follow-up (*P* < 0.05) (Table 6).

#### 3.2.5. Comparison of Different Degrees of Mood Disorder

To further investigate the effects of BSSG-P and BSSG on menopausal patients with different degrees of mood disorder, statistical analysis was performed separately for the patients with moderate and serious mood disorder.

For the patients with moderate mood disorder, there was no significant difference in Greene, SDS and SAS before treatment between BSSG-P and BSSG groups, indicating that the two groups have comparability (*P* > 0.05). Effects of lowering the score of Greene, SDS, and SAS were more remarkable in the BSSG-P group than in the BSSG group at the end of the 8th and 12th weeks (*P* < 0.05), whereas there was no significant difference between groups after 4 weeks of treatment (*P* > 0.05). Refer to the mean difference of Greene, SDS, and SAS before and after treatment between groups, BSSG-P group was better than BSSG group at the end of the 8th and 12th week except the change of SAS after 8 weeks of treatment (*P* < 0.05), although reduction of score of SAS in BSSG-P group was more pronounced (*P* = 0.06). There was no significant difference in Greene, SDS, and SAS at the end of the 4th week between groups (*P* > 0.05).

For the patients with serious mood disorder, neither comparison between groups nor comparison of mean difference between groups had statistical significance at the end of the 4th, 8th, and 12th week (*P* > 0.05). These results showed that there was no significant difference between BSSG-P and BSSG in the treatment of patients with serious mood disorder. As shown in Table 7.

#### 3.2.6. Effect of BSSG-P and BSSG on Dopamine (DA) and Norepinephrine (NA)

We found that both BSSG-P and BSSG could raise the levels of DA and NA after treatment (*P* < 0.05). However, the difference was not statistically different before and after treatment between groups as well as was comparison of mean difference between groups (*P* > 0.05) (Table 8).

#### 3.2.7. Safety Analysis of BSSG-P and BSSG

Routine examinations found no abnormalities in urine, blood, liver function, renal function, and electrocardiogram at the end of the 8th week in both two groups.

During treatment, one participant in BSSG-P group, who had no ovarian cyst before treatment, developed ovarian cyst with a diameter of less than 3 cm after 8 weeks' treatment. One patient had a left ovarian cyst (22 mm × 23 mm) before treatment and became a right ovarian cyst (20 mm × 14 mm) after treatment, and one case of ovarian cyst disappeared after treatment. In the BSSG group, one patient developed ovarian cyst with a diameter of less than 3 cm after intervention. The patients with ovarian cyst after treatment still had menstruation, and there was no difference between the two groups.

#### 3.2.8. Adverse Events and Adherence

There were no statistically significant differences between BSSG-P group and BSSG group in the proportion of participants with adverse events during the 8 weeks' treatment (2 in BSSG-P and 2 in BSSG), and this was examined by chi-square test (*P* = 0.98), as identified by experts, the adverse events had nothing to do with medication. 85 cases were included in this study, of which 13 participants were lost or terminated in the course of treatment, with a total dropout rate of 15%. The main complaints from participants include diarrhea, dizziness/discomfort in stomach (2 in BSSG and 2 in BSSG-P), lack of symptom relief (2 in BSSG-P and 5 in BSSG), fracture of ribs in traffic accident (1 in BSSG), and personal reasons--residing abroad (1 in BSSG-P).

## 4. Discussion

Menopause is a phase of life when women experience both physical and mental changes with a considerable variety of symptoms, which will last for a long period of time accompanied with different symptoms at different stages. The HT treatment is currently emphasizing individualized therapy, thus the overall concept of TCM and syndrome differentiation and treatment should fully reflect the combination of commonness and individualization, and accordingly effective treatment should be developed to tackle the problems for different symptoms and stages of menopausal syndrome women.

TCM has been used in China for thousands of years and has a pivotal role in the prevention and treatment of diseases and posttreatment recovery [26]. Although clinical and experimental studies have shown that Chinese herbal formula is superior to placebo in the treatment of postmenopausal women's symptoms [27–29], there is little evidence that TCM could improve the psychosocial symptoms in the perimenopausal and postmenopausal women at present [30]. TCM emphasizes the integration of the form and the spirit, therefore, besides Chinese herbal medicine, other TCM methods of comprehensive treatment like psychotherapy, acupuncture and tai chi are employed in this field [31]. Different from modern psychology, TCM-based psychotherapy, with thousands of years of history in China, has the characteristics of oriental speculation, development, personality differences, and overall regulation, which has a good effect on mental disorder. Menopausal women, who are in a critical period of physiological and social role transition, are prone to suffer from psychosomatic diseases due to agitation or confusion. Thus, both dialectical use of Chinese herbal medicine and application of TCM-based psychotherapy should be fully involved in treatment for mood disorder in menopausal women on the basis of the holistic concept of “the integration of the form and the spirit”.

In our previous clinical studies, BSSG-P has been shown to be superior to BSSG, placebo, and psychological treatment in improving perimenopausal syndrome [19]. In this study, we improved and optimized the psychosomatic treatment of BSSG-P with the aim of producing obvious curative effect in treating patients with moderate to serious disorder. The results showed that for all patients, the scores of Greene, SDS and SAS were significantly decreased in both BSSG-P and BSSG groups at the end of the 4th, 8th, and 12th week, indicating that both BSSG-P and BSSG had obvious effect and advantage in improving menopausal patients with mood disorder. However, both BSSG-P and BSSG failed to obviously improve sexual dimension. Similarly, no significant effect was observed for HT on the improvement of sexual function in perimenopausal women [32]. A systematic review has observed that HT had certain positive effect on sexual function possibly due to the improvement of vaginal dryness [33]. Anyway, there is a lack of strong evidence and long-term safety data to prove that HT can improve sexual function [34].

At the end of the 4th week, there was no significant difference between BSSG-P and BSSG in improving the mood disorder in menopausal women. The advantage of BSSG-P was obvious at the end of the 8th week of treatment and the 12th week of follow-up. Thus, we believed that BSSG-P had better overall curative effect, and the efficacy was more comprehensive and stable when compared with BSSG. The mood disorder in menopausal women is regarded as a chronic disease. Both BSSG and BSSG-P need a period of time to be effective, followed by a gradual and orderly progress. So in the initial stage we needed to stick to treatment, and did not give up because of the initial failure. The obvious effect of BSSG-P treatment after 8 weeks of treatment indicated that for patients with moderate to serious mood disorder, BSSG-P could ameliorate the physical symptoms in the early stage of treatment to improve the psychological symptoms. However, it failed to exert therapeutic effect with the extension of treatment time as mood disorder of menopausal syndrome is a psychosomatic disease. In this case, the superiority and importance of BSSG-P were in full view.

According to different degrees of mood disorders, the effect of BSSG-P was obviously better than that of BSSG in patients with moderate mood disorder. However, no significant difference was observed in patients with serious mood disorder, which might be due to the small sample size. Large-scale randomized clinical trial is required to further evaluate the potential advantages of BSSG-P. Besides, TCM emphasizes individual syndrome differentiation and treatment, it may be related to the same psychosomatic therapeutic schedule for patients with different degrees of mood disorders, however seldom special plan for those with serious mood disorder has been carried out. Patients with serious mood disorder not only have too long course of disease to achieve efficacy in the short term, but also have complex clinical manifestations and often expect too much. Behavioral characteristics, such as the degree of coordination in therapy, the depth of conversation with the therapist, and cognitive characteristics, such as the degree of cognitive impairment, and the level of understanding of therapy, are also critical to the therapeutic effect. In this paper, we improved the previous emotional therapy for only 4 weeks, and obtained good results for patients with moderate mood disorder. Therefore, it is necessary to further improve the emotional therapeutic regimen for patients with serious mood disorder, and increase the treatment course and times of BSSG-P to assess its clinical efficacy.

In this study, both BSSG-P and BSSG significantly increased the levels of DA and NA after treatment within groups, which suggested that the mechanism of BSSG-P and BSSG to improve moderate to serious mood disorder in menopausal period may be associated with the increase of the content of DA and NA of monoamine neurotransmitters. However, no statistically significant difference was found between groups, although the BSSG-P group showed superiority after 8-week treatment, which might be due to the small sample size. A large sample size is required to further observe the potential difference between the two groups.

## 5. Conclusions

Both BSSG and BSSG-P treatment could effectively improve the physical and psychological symptoms of menopausal women with mood disorder and had favorable safety profile. The effect of BSSG-P was superior to that of BSSG. For patients with moderate mood disorder, BSSG-P treatment showed obvious advantage, especially in improving the symptoms and depression. However, no advantage was observed in patients with serious mood disorder. Therefore, more strictly designed large-scale randomized clinical trials are needed to evaluate the efficacy of BSSG-P in menopausal patients with serious mood disorder.

## Figures and Tables

**Table 1:** Baseline demographic and clinical characteristics of the participants.

Characteristic	BSGG (n=42)	BSSG-P (n=43)	*P*
Mean age (SD), year	50.44±4.70	51.91±3.83	0.16
Mean height (SD), cm	159.47±6.29	158.15±4.63	0.33
Mean weight (SD), KG	53.09±8.35	54.26±8.55	0.57
Menarche (SD), year	13.50±1.11	13.32±1.27	0.18

Occupational distribution n (%)	Farmer, 1 (2.4); Administrative worker, 4 (9.5); Intellectual, 6 (14.3); Ordinary workers, 6 (14.3); Freelancers, 8 (19.0); Other, 17 (40.5)	Farmer, 1 (2.3); Administrative worker, 3 (7.0); Intellectual, 9 (20.9); Ordinary workers, 6 (14.0); Freelancers, 5 (11.6); Other, 19 (44.2)	0.93^#^

Marriage, n (%)	Unmarried, 0 (0); Married, 36 (85.7); Remarried, 2 (4.8); Divorced, 3 (7.1); Widowed, 1 (2.4)	Unmarried, 0 (0); Married, 38 (88.4); Remarried, 2 (4.65); Divorced, 2 (4.65); Widowed, 1 (2.3)	0.40^#^

Working pressure, n (%)	No/Small, 20 (47.6); Moderate, 18 (42.9); Great, 4 (9.5)	no/small, 18 (41.9); Moderate, 22 (51.2); great, 3 (6.9)	0.73
Smoking, n (%)	No, 42 (100); Yes, 0 (0)	No, 42 (97.7); Yes, 1 (2.3)	1.00^#^
Drinking, n (%)	No, 39 (92.9); Yes, 3 (7.1)	No, 41 (95.35); Yes, 2 (4.65)	0.68^#^
Coffee, n (%)	No, 39 (92.9); Yes, 3 (7.1)	No, 38 (88.4); Yes, 5 (11.6)	0.72^#^
Menstrual cycle, n (%)	Normal, 6 (14.3); Abnormal, 36 (85.7)	Norma, l7 (16.3); Abnormal, 36 (83.7)	0.80
Duration of each cycle n (%)	Normal, 10 (23.8); Abnormal, 32 (76.2)	Normal, 8 (18.6); Abnormal, 35 (81.4)	0.56
Dysmenorrhea, n (%)	No, 16 (38); Yes, 26 (62)	No, 19 (44.2); Yes, 24 (55.8)	0.57
Disease status, n (%)	≤1 y, 23 (54.7); 1-3 y, 12 (4.8); ≥3 y, 7 (40.5)	≤1 y, 18 (41.9); 1-3 y, 17 (39.5); ≥3 y, 8 (18.6)	0.75

# Values are calculated by Fisher's Exact Test.

**Table 2:** Comparison of the baseline data in Greene, SDS, SAS, and all dimensions.

	Dimensions	BSSG (n=42)	BSSG-P (n=43)	t/z	*P*
Greene	Total	32.62±6.16	33.68±6.46	0.65	0.52
	Anxiety	11.00±2.10	10.97±2.32	0.06	0.96
	Depression	8.00±2.63	8.09±2.40	0.15	0.88
	Somatic	8.03±3.18	9.44±3.53	1.66	0.10
	Vascular	3.48±1.71	3.12±1.60	0.86	0.39^#^
	Sexual	2.16±0.74	2.19±0.69	0.04	0.97^#^

SDS	Total	51.62±6.28	52.35±6.45	0.45	0.66
	Anxiety	20.39±2.50	19.94±2.72	0.72	0.48^#^
	Self-loss	11.19±3.02	11.47±3.36	0.34	0.73
	Motivation lack	12.45±1.59	12.41±2.11	0.36	0.72^#^
	Physiological symptom	8.90±1.81	9.34±2.09	0.54	0.59^#^

SAS	Total	51.48±5.95	52.03±6.26	0.35	0.73
	Anxiety	10.74±1.81	11.06±1.72	0.78	0.43^#^
	The autonomic nervous disorder	9.58±1.67	9.31±1.94	0.62	0.54^#^
	Somatic	17.90±2.82	18.03±2.88	0.22	0.82^#^
	Exercise tension dimension	5.65±1.56	5.62±1.66	0.10	0.92^#^

# is the rank sum test, and the statistical value is Z value

**Table 3:** Comparison of the total score of Greene and the scores of each dimension.

Outcome variables Greene		Comparison before and after treatment within groups at each time point						Comparison between groups at each time point	
		BSSG (n=39)			BSSG-P (n=39)				
		$\overset{-}{x}\pm s$	*t/z*	*P*	$\overset{-}{x}\pm s$	*t/z*	*P*	*t/z*	*P*
Total	Baseline	32.62±6.16			33.68±6.46				
	4 weeks	25.31±5.49	6.45	<0.01	22.45±7.20	7.36	<0.01	1.72	0.79
	week	23.59±6.98	4.42	<0.01^#^	18.48±6.85	9.66	<0.01	2.86	0.01
	12 weeks	25.21±5.82	5.73	<0.01	20.90±6.27	8.74	<0.01	2.75	0.01
Anxiety,	Baseline	11.00±2.10			10.97±2.32				
	4 weeks	8.55±2.47	4.49	<0.01	7.50±2.54	6.65	<0.01	1.70	0.90
	8 weeks	8.42±2.50	5.29	<0.01	6.22±2.84	7.78	<0.01	3.26	<0.01
	12 weeks	8.90±2.32	3.63	<0.01^#^	7.22±2.41	6.60	<0.01	2.83	<0.01
Depression	Baseline	8.00±2.63			8.09±2.40				
	4 weeks	6.42±2.70	3.40	<0.01	5.59±3.06	4.04	<0.01	1.39	0.16^#^
	8 weeks	5.58±2.62	4.91	<0.01	4.00±2.57	6.97	<0.01	2.57	0.01^#^
	12 weeks	6.42±2.60	3.43	<0.01	4.28±1.82	7.52	<0.01	3.80	<0.01
Somatic	Baseline	8.03±3.18			9.44±3.53				
	4 weeks	6.39±2.28	3.22	<0.01	5.91±2.67	6.03	<0.01	0.56	0.57^#^
	8 weeks	6.55±2.67	2.86	<0.01	5.22±3.31	6.73	<0.01	1.96	0.05^#^
	12 weeks	6.77±2.78	2.05	0.05	5.34±2.54	4.46	<0.01^#^	2.02	0.04^#^
Vascular	Baseline	3.48±1.71			3.12±1.60				
	4 weeks	2.45±1.36	3.50	<0.01	1.97±1.56	2.91	<0.01^#^	1.70	0.09^#^
	8 weeks	2.29±1.30	4.59	<0.01	1.69±1.38	5.50	<0.01	1.83	0.07^#^
	12 weeks	2.71±1.30	1.89	0.06^#^	2.00±1.19	2.71	<0.01^#^	1.97	0.05^#^
Sexual	Baseline	2.16±0.74			2.19±0.69				
	4 weeks	2.13±0.50	0.72	0.47^#^	1.88±0.55	1.95	0.05^#^	1.82	0.07^#^
	8 weeks	2.00±0.52	1.57	0.12^#^	2.03±0.70	1.09	0.28^#^	0.03	0.97^#^
	12 weeks	2.16±0.52	0.46	0.64^#^	1.91±0.59	1.72	0.09^#^	1.72	0.09^#^

# is the rank sum test, and the statistical value is Z value.

**Table 4:** Comparison of the total score of SDS and the scores of each dimension.

Outcome variable SDS		Comparison before and after treatment within groups at each time point						Comparison between groups at each time point	
		BSSG (n=39)			BSSG-P (n=39)				
		$\overset{-}{x}\pm s$	*t/z*	*P*	$\overset{-}{x}\pm s$	*t/z*	*P*	*t/z*	*P*
Total	Baseline	51.62±6.28		<0.01	52.35±6.45				
	4 weeks	42.90±5.92	8.73	<0.01	41.65±6.23	9.50	<0.01	0.80	0.43
	8 weeks	42.10±6.42	8.24	<0.01	37.48±6.02	12.42	<0.01	2.88	0.01
	12 weeks	43.52±6.46	4.12	<0.01^#^	38.52±6.18	11.37	<0.01	3.07	<0.01
Anxiety	Baseline	20.39±2.50			19.94±2.72				
	4 weeks	17.10±3.35	3.96	<0.01^#^	15.75±3.12	6.74	<0.01	1.65	0.10
	8 weeks	16.71±3.00	7.20	<0.01	14.06±2.26	10.84	<0.01	3.96	<0.01
	12 weeks	17.23±2.18	6.44	<0.01	14.84±2.42	8.69	<0.01	3.50	<0.01
Self-loss	Baseline	11.19±3.02			11.47±3.36				
	4 weeks	9.13±2.92	4.69	<0.01	9.16±2.90	4.73	<0.01	0.00	1.00^#^
	8 weeks	8.94±2.86	4.80	<0.01	8.19±2.57	5.54	<0.01	1.20	0.23^#^
	12 weeks	9.55±2.59	3.84	<0.01	7.94±2.17	6.05	<0.01	2.36	0.02
Motivation lack	Baseline	12.45±1.59			12.41±2.11				
	4 weeks	8.29±1.51	6.50	<0.01^#^	8.09±1.33	6.07	<0.01^#^	0.63	0.53^#^
	8 weeks	11.13±3.49	4.08	<0.01^#^	9.47±1.83	6.75	<0.01^#^	2.34	0.02^#^
	12 weeks	11.29±4.10	3.19	<0.01^#^	10.75±5.55	4.32	<0.01^#^	1.72	0.08
Physiological	Baseline	8.90±1.81			9.34±2.09				
	4 weeks	7.00±1.73	5.08	<0.01	6.66±1.62	4.83	<0.01^#^	0.81	0.42
	8 weeks	6.94±1.93	4.98	<0.01	6.31±1.60	5.26	<0.01^#^	1.30	0.19^#^
	12 weeks	7.45±2.08	2.63	<0.01^#^	6.66±1.49	4.94	<0.01^#^	1.66	0.10

Data were expressed as mean. ^#^ is the rank sum test, and the statistical value is Z value.

**Table 5:** Comparison of the total score of SDS and the scores of each dimension.

Outcome variable SAS		Comparison before and after treatment within groups at each time point						Comparison between groups at each time point	
		BSSG (n=39)			BSSG-P (n=39)				
		$\overset{-}{x}\pm s$	*t/z*	*P*	$\overset{-}{x}\pm s$	*t/z*	*P*	*t/z*	*P*
Total	Baseline	51.48±5.95			52.03±6.26				
	4 weeks	40.48±5.59	5.29	<0.01^#^	39.29±6.90	9.50	<0.01	**0.80**	**0.43**
	8 weeks	39.76±5.77	10.12	<0.01	36.39±5.90	12.42	<0.01	2.88	0.01
	12 weeks	41.34±5.75	8.94	<0.01	37.77±5.73	11.37	<0.01	3.07	<0.01
Anxiety	Baseline	10.74±1.81			11.06±1.72				
	4 weeks	8.35±2.17	6.78	<0.01	8.25±2.34	4.91	<0.01^#^	1.65	0.10
	8 weeks	8.00±1.95	7.90	<0.01	6.84±1.59	12.59	<0.01	3.96	<0.01
	12 weeks	8.29±1.72	4.57	<0.01^#^	7.25±1.65	5.90	<0.01^#^	3.50	<0.01
Autonomic nervous disorder									
	Baseline	9.58±1.67			9.31±1.94				
	4 weeks	7.81±1.80	3.65	<0.01^#^	7.41±1.64	3.99	<0.01^#^	0.00	1.00^#^
	8 weeks	7.77±1.96	4.07	<0.01	6.78±1.70	4.95	<0.01	1.20	0.23^#^
	12 weeks	8.48±1.71	3.27	<0.01	7.50±1.41	4.09	<0.01	2.36	0.02
Somatic	Baseline	17.90±2.82			18.03±2.88				
	4 weeks	14.61±2.40	4.17	<0.01^#^	13.72±3.20	7.23	<0.01	0.63	0.53^#^
	8 weeks	14.71±2.76	7.16	<0.01	13.16±3.11	7.74	<0.01	2.34	0.02^#^
	12 weeks	14.65±2.63	7.29	<0.01	13.56± 2.85	6.67	<0.01	1.72	0.08
Exercise tension	Baseline	5.65±1.56			5.62±1.66				
	4 weeks	4.65±1.31	4.31	<0.01	4.38±1.34	5.46	<0.01	0.81	0.42
	8 weeks	4.55±1.26	3.13	<0.01^#^	4.03±1.49	4.25	<0.01	1.30	0.19^#^
	12 weeks	4.84±1.27	3.01	<0.01	4.50±1.30	3.55	<0.01	1.66	0.10

Data were expressed as mean. ^ #^ is the rank sum test, and the statistical value is Z value.

**Table 6:** Mean difference between time points and pretreatment was compared between groups.

Outcome variable	Time points (week)	BSSG (n=39)	BSSG-P (n=39)	*t/z*	*P*
Greene	8 w	9.03±6.41	15.19±8.76	2.56	0.01^#^
	12 w	7.41±6.97	12.77±8.14	2.73	0.01
SDS	8 w	9.52±6.22	14.87±6.67	3.21	0.02
	12 w	8.10±6.51	13.84±6.78	3.28	<0.01^#^
SAS	8 w	11.72±6.24	15.65±7.89	2.13	0.04
	12 w	10.14±6.11	14.26±7.38	2.35	0.02

Data were expressed as mean. ^#^ is the rank sum test, and the statistical value is Z value.

**Table 7:** Greene, SDS, and SAS score for moderate mood disorder and serious mood disorder, respectively.

Outcome variable (week)		Moderate mood disorder (before and after treatment between groups)								Serious mood disorder (before and after treatment between groups)							
		Comparison				Comparison of mean difference				Comparison				Comparison of mean difference			
		BSSG (n=34)	BSSG-P (n=30)	*t*	*P*	BSSG (n=34)	BSSG-P (n=30)	* t*	*P*	BSSG (n=5)	BSSG-P (n=9)	*t*	*P*	BSSG (n=5)	BSSG-P (n=9)	*t*	*P*
		$\overset{-}{x}\pm s$	$\overset{-}{x}\pm s$			$\overset{-}{x}\pm s$	$\overset{-}{x}\pm s$			$\overset{-}{x}\pm s$	$\overset{-}{x}\pm s$			$\overset{-}{x}\pm s$	$\overset{-}{x}\pm s$		
Greene,	0 w	31.25±1.06	32.78±6.52	0.89	0.38					39.20±3.02	35.00±1.93	1.23	0.25				
	4 w	25.04±1.20	21.43±1.49	1.89	0.07	6.21±1.45	11.35±1.99	1.63	0.10	26.60±1.44	23.43±1.72	1.33	0.21	12.60±2.68	11.57±1.72	0.34	0.74
	8 w	22.79±1.48	16.74±0.25	3.12	<0.01	8.45±1.332	16.04±9.15	3.29	<0.01	27.40±1.89	23.43±2.87	1.05	0.32	11.80±2.58	11.57±2.76	0.06	0.96
	12 w	24.79±1.23	19.17±1.19	3.27	<0.01	6.46±1.22	13.61±1.88	3.22	<0.01	27.20±2.04	26.00±2.14	0.39	0.70	12.00±4.51	9.00±0.95	0.77	0.46
SDS,	0 w	49.88±1.08	49.91±0.91	0.03	0.98					60.00±1.45	60.71±2.18	0.25	0.81				
	4 w	42.21±1.25	40.17±1.26	1.15	0.26	7.67±0.10	9.74±1.39	1.22	0.23	46.20±1.59	46.43±1.95	0.09	0.93	13.80±2.31	14.29±1.41	0.19	0.85
	8 w	41.67±1.42	35.09±0.84	3.95	0.00	8.21±1.17	14.83±1.75	3.58	<0.01	44.20±1.02	45.00±2.14	0.30	0.77	15.80±4.87	15.71±2.42	0.03	0.89
	12 w	42.75±1.34	35.87±0.89	4.26	0.00	7.12±1.21	14.04±1.51	3.59	<0.01	47.20±2.25	46.86±1.50	0.13	0.90	12.80±3.50	13.80±2.18	0.27	0.79
SAS,	0 w	50.92±1.02	51.04±1.08	0.09	0.93					54.20±4.31	53.43±2.99	0.15	0.88				
	4 w	39.88±1.05	38.61±1.50	0.70	0.49	11.04±1.07	12.44±1.46	0.77	0.44	43.40±3.27	39.71±1.69	1.09	0.30	10.80±3.10	13.71±2.21	0.79	0.45
	8 w	39.42±1.26	35.61±1.34	2.07	0.04	11.50±1.27	15.44±1.61	1.93	0.06	41.40±1.50	39.14±1.22	1.17	0.27	12.80±3.15	14.28±2.86	0.34	0.74
	12 w	41.17±1.14	36.39±1.21	2.88	0.01	9.75±1.28	14.65±1.45	2.53	0.02	42.20±3.23	42.43±1.02	0.08	0.94	12.00±2.39	11.00±2.66	0.27	0.80

Data were expressed as mean. “Comparison” refers to comparison of each time point and 0 week between groups. “Comparison of mean difference” means a comparison of differences between each time point and 0 week between groups

**Table 8:** Effect of BSSG-P and BSSG on DA and NA.

Ng/ml	Comparison within groups								Between groups							
									Comparison				Comparison of mean difference			
	BSSG (n=36)				BSSG-P (n=38)				BSSG (n=36)	BSSG-P (n=38)	*t/z*	*P*	BSS (n=36)	BSSG-P (n=38)	*t/z*	*P*
	Pre-T	Post-T	*t/z*	*P*	Pre-T	Post-T	*t/z*	*P*								
									$\overset{-}{x}\pm s$	$\overset{-}{x}\pm s$			$\overset{-}{x}\pm s$	$\overset{-}{x}\pm s$		
DA	3.95±2.3	5.31±2.18	2.35	0.02	4.41±3.67	6.71±4.87	2.03	0.04^#^	5.31±2.18	6.71±4.87	0.16	0.88^#^	1.36±1.21	2.30±1.59	0.33	0.74^#^
NA	144.28±35.10	278.74±53.91	2.68	0.03	149.68±31.04	298.65±73.33	3.12	0.02	278.74±53.91	298.65±73.33	0.22	0.83	134.40±44.10	148.90±49.35	0.22	0.83

^#^ is the rank sum test, and the statistical value is Z value. Pre-T means pretreatment; Post-T means posttreatment. “Comparison” refers to comparison of each time point and baseline between groups. “Comparison of mean difference” means a comparison of differences between each time point and baseline between groups.

## Data Availability

The data used to support the findings of this study are available from the corresponding author upon request.
